# Advantages of enhanced recovery after surgery program in robot-assisted radical cystectomy

**DOI:** 10.1038/s41598-023-43489-w

**Published:** 2023-09-27

**Authors:** Masaki Nakamura, Ibuki Tsuru, Taro Izumi, Akihiro Ono, Yasushi Inoue, Yasuko Muraki, Yumi Yamada, Yuko Tsuji, Junko Watanabe, Mutsuko Fujimura, Shunsuke Kihara, Akihiro Naito, Taichi Shiratori, Ryo Amakawa, Hiroki Inatsu, Tadashi Yoshimatsu, Masanori Kashiwagi, Akira Fukuda, Teppei Morikawa, Masashi Kusakabe, Motofumi Suzuki, Shuji Kameyama, Haruki Kume, Yoshiyuki Shiga

**Affiliations:** 1 Department of Urology, NTT Medical Center Tokyo, 5-9-22, Higashigotanda, Shinagawa-Ku, Tokyo, 141-8625 Japan; 2 Information System Group, NTT Medical Center Tokyo, Tokyo, Japan; 3 Critical Pathway Committee, NTT Medical Center Tokyo, Tokyo, Japan; 4 Nursing Department, NTT Medical Center Tokyo, Tokyo, Japan; 5 Nutrition Department, NTT Medical Center Tokyo, Tokyo, Japan; 6 Department of Rehabilitation, NTT Medical Center Tokyo, Tokyo, Japan; 7 Operating Department, NTT Medical Center Tokyo, Tokyo, Japan; 8 Department of Urology, Tokyo Metropolitan Bokutoh Hospital, Tokyo, Japan; 9 Department of Anesthesiology, NTT Medical Center Tokyo, Tokyo, Japan; 10 Department of Diagnostic Pathology, NTT Medical Center Tokyo, Tokyo, Japan; 11 Department of Radiology, NTT Medical Center Tokyo, Tokyo, Japan; 12 Tokyo Healthcare University, Tokyo, Japan; 13 Department of Urology, The University of Tokyo Hospital, Tokyo, Japan

**Keywords:** Bladder, Surgical oncology

## Abstract

Radical cystectomy is a gold-standard treatment for muscle-invasive bladder cancer. We recently introduced robot-assisted radical cystectomy (RARC) with perioperative enhanced recovery after surgery (ERAS). The medical records of patients with bladder cancer who underwent open radical cystectomy (ORC) or RARC/ERAS at NTT Medical Center Tokyo were retrospectively reviewed to compare the surgical outcomes, hospital stay, and medical costs between groups. Multidisciplinary full ERAS items were provided for the RARC/ERAS group. The median estimated blood losses in the ORC and RARC/ERAS groups were 650 and 100 mL, and the median operative times were 312 and 445 min, respectively. In addition, the median times to liquid food intake in these groups were 6 and 0 days, the median times to first flatus and first defecation were 2 and 1 day, and 3 and 1.5 days, respectively. The rates of postoperative ileus in the ORC and RARC/ERAS groups were 27.5% and 4.5%, and the median postoperative hospital stays was 26.5 and 12 days, respectively. Medical costs excluding surgery were significantly lower in the RARC/ERAS group. In conclusion, RARC/ERAS represents a safe treatment option for muscle-invasive bladder cancer with decreased perioperative complications and lower medical costs.

## Introduction

Bladder cancer is the 10th most common cancer globally. In Japan, more than 9000 patients died of bladder cancer in 2022^1^. Tobacco smoking, exposure to carcinogens including organic solvents, male sex, and advanced age are well-known risk factors for bladder cancer^2,3^.

Radical cystectomy with urinary diversion is the gold-standard surgical treatment for muscle-invasive bladder cancer and carcinoma in situ, which are refractory to bacillus Calmette-Guérin intravesical immunotherapy. Urinary diversions after radical cystectomy include ileal conduit, ileal neobladder, and ureterostomy. For urinary diversion using the small intestine in particular, the surgical procedure can be especially invasive.

Although robot-assisted radical cystectomy (RARC) has been replacing open radical cystectomy (ORC) or laparoscopic radical cystectomy in Japan since it gained insurance coverage in 2018, reports on RARC with enhanced recovery after surgery (ERAS) are rare. In this study, we report our early experience with RARC/ERAS, and surgical outcomes and medical costs were compared between RARC/ERAC and ORC. The objective of this retrospective observational study was to verify the advantages of RARC/ERAS concerning postoperative patient recovery and medical costs.

## Results

In total, 40 patients who underwent ORC and 22 patients who underwent RARC/ERAS were enrolled in this retrospective observational study. Patient characteristics at baseline are presented in Table 1. The median patient ages in the ORC and RARC/ERAS groups were 74 and 75.5 years, respectively, the proportions of men were 80.0% and 90.9%, respectively, and the BMIs were 21.3 and 21.8 kg/m^2^, respectively. These variables did not differ between the groups. Neoadjuvant chemotherapy (gemcitabine plus cisplatin) was administered in 17 patients in the ORC group and 11 patients in the RARC/ERAS group. Preoperative hospital stay was significantly longer in the ORC group because of the need for preoperative mechanical bowel preparation (4.5 days vs 1 day, p < 0.001). As part of the ERAS protocol, patients in the RARC/ERAS group received preoperative stoma care training by a certified nurse.

**Table 1:** Patient characteristics.

Variables	ORC	RARC/ERAS	*P*
No. of patients	40	22	
Median age, years (IQR)	74.0 (66.5–79.3)	75.5 (67.3–78.0)	0.721
Male sex, n (%)	35 (85.4)	20 (90.9)	0.702
Median BMI, kg/m^2^ (IQR)	21.3 (18.9–25.3)	21.8 (20.2–24.4)	0.588
NAC, yes/no	17/23	11/11	0.792
Preoperative stays, days (IQR)	4.5 (3.75–5)	1 (1–3)	< 0.001

*BMI* body mass index, *ERAS* enhanced recovery after surgery, *IQR* interquartile range, *ORC* open radical cystectomy, *RARC* robot-assisted radical cystectomy.

The surgical results are presented in Table 2. The median estimated blood loss was significantly smaller in the RARC/ERAS group, whereas the operative time was longer in this group. In the ORC group, 17.5% of patients received blood transfusions. The median numbers of retrieved lymph nodes were 10 and 22 in ORC and RARC/ERAS groups, respectively (*P* < 0.001). Because we performed ERAS simultaneously with RARC, ERAS was not adopted in the ORC group. Patients in the RARC/ERAS group started fluid intake, liquid food intake, and mobilization at 3 h after surgery. Notably, the median times to first flatus and first defecation were significantly shorter in the RARC/ERAS group. Concerning complications, postoperative paralytic ileus (POI) developed in 27.5% and 4.5% of patients in the ORC and RARC/ERAS groups, respectively. The length of hospital stay was significantly shorter in the RARC group than in the ORC group (12 days vs. 26.5 days).

**Table 2:** Surgical results and postoperative characteristics.

Variables	ORC (n = 40)	RARC/ERAS (n = 22)	*P*
Median eBL, mL (IQR)	650 (517–856)	100 (50–150)	< 0.001
Median operative time, min (IQR)	312 (274–333)	445 (391–504)	< 0.001
Median LN yield, n (IQR)	10 (7.75–11.75)	22 (17–26)	< 0.001
Urinary diversion, n (%)			0.397
Ileal conduit	35 (87.5)	20 (91.0)	
Neobladder	2 (5.0)	1 (4.5)	
Ureterostomy	3 (7.5)	1 (4.5)	
Blood transfusion, n (%)	7 (17.5)	0 (0)	0.02
ERAS adaptation, n (%)	0 (0)	22 (100)	< 0.001
Median time to fluid intake, days (IQR)	3 (2–4)	0 (0–0)	< 0.001
Median time to liquid food intake, days (IQR)	6 (4–8.5)	0 (0–0)	< 0.001
Median time to mobilization, days (IQR)	1 (1–1)	0 (0–0)	0.223
Median time to first flatus, days (IQR)	2 (1–3)	1 (0–1)	0.008
Median time to first defecation, days (IQR)	3 (2–5)	1.5 (1–2)	< 0.001
Postoperative complication within 30 days			
POI, n (%)	11 (27.5)	1 (4.5)	0.028
Clavien–Dindo grade > 3b, n (%)	4 (10)	0 (0)	< 0.001
Median postoperative hospital stay, days (IQR)	26.5 (22–37.3)	12 (10–15)	< 0.001

*eBL* estimated blood loss, *ERAS* enhanced recovery after surgery, *IQR* interquartile range, *LN* lymph node, *ORC* open radical cystectomy, *POI* postoperative paralytic ileus, *RARC* robot-assisted radical cystectomy.

Medical costs were compared between the groups (Table 3). Drug fees including oral medication and injections were decreased by nearly fivefold in the RARC/ERAS group (14.2 thousand yen vs. 78.7 thousand yen). Examination fees including test and diagnostic imaging costs also were lower in the RARC/ERAS group (58.9 thousand yen vs. 122.9 thousand yen). The operation/anesthesia fees were 1513.9 and 1658.9 thousand yen in the ORC and RARC/ERAS groups, respectively, reflecting the higher insurance fee point for RARC. Medical fees excluding operation/anesthesia were 964.7 and 655.7 thousand yen in the ORC and RARC/ERAS groups, respectively. In total, the medical costs in these groups were 2478.6 and 2314.6 thousand yen, respectively.

**Table 3:** Comparison of medical costs between ORC and RARC/ERAS (× 10^3^ JPY).

Category	ORC	RARC/ERAS	*P*
Drug cost, median (IQR)	78.7 (36.3–125.4)	14.2 (11.9–22.4)	< 0.001
Operation/anesthesia cost, median (IQR)	1513.9 (1433.3–1634.1)	1658.9 (1522.8–1715.0)	0.041
Hospitalization cost, median (IQR)	788.4 (653.6–929.4)	557.8 (521.9–656.5)	0.009
Examination cost, median (IQR)	122.9 (78.7–185.5)	58.9 (53.1–71.1)	< 0.001
Total cost, median (IQR)	2478.6 (2282.7–2992.0)	2314.6 (2150.2–2450.6)	< 0.001

*ERAS* enhanced recovery after surgery, *JPY* Japanese yen, *IQR* interquartile range, *ORC* open radical cystectomy, *RARC* robot-assisted radical cystectomy.

## Discussion

The prognosis of muscle-invasive bladder cancer is poor. Even with improvements in multimodal treatments including radical cystectomy and chemotherapy, the 12-month disease-free survival remains as low as 62.8%^4^. In multimodal treatment for bladder cancer, it is important to reduce the invasiveness of surgery. Despite the progress in surgical techniques, radical cystectomy remains associated with a considerably high risk of complications^5^.

POI is one of the major postoperative complications after radical cystectomy, occurring at the incidence of 1.58–23.5%^6–8^. Older age and higher BMI are associated with the development of POI^8^. POI has mainly been studied in patients undergoing gastrointestinal surgeries^7,9–12^. Gum chewing and early oral fluid intake alone have not prevented POI^12,13^. In the ERAS protocol, several items for the prevention of POI including carb loading, the prevention of postoperative nausea and vomiting (PONV), opioid-sparing analgesia, and early mobilization are applied. The synergistic effect of these strategies might greatly reduce the risk of POI. In this study, the incidence of POI was significantly lower in the RARC/ERAS group than in the ORC group. Notably, the patient with POI in the RARC/ERAS group had comorbid hypothyroidism and required 100 IU of levothyroxine sodium hydrate. Delayed replacement of levothyroxine sodium hydrate during fasting might have led to impaired intestinal function.

ERAS was first reported as a multidisciplinary approach to facilitate postoperative recovery in gastrointestinal surgeries^14^. A meta-analysis of colorectal surgeries revealed significant advantage of ERAS in reducing the complication rate (relative risk = 0.53, 95% confidence interval = 0.44–0.64) and shortening the length of hospital stay (− 2.55 days)^15^. Preferable effects of ERAS protocol during open radical cystectomies have been reported by several groups^16–18^. Notably, a randomized controlled trial clarified lower rates of postoperative complications and lower demand for analgesics in the ERAS group compared to the conservative regimen group^17^. In this setting, however, there were no significant differences between the groups in terms of gastrointestinal events^17^.

Few reports investigate the effect of ERAS protocol during RARC. A comparative study reported that a multifactorial fast-track regimen decreased time to mobilization, time to regular diet, and the use of postoperative morphine equivalents in the RARC setting^19^. Another retrospective study reported a shorter length of hospital stay in the enhanced recovery program group during RARC^20^. Although the evidence levels were low, the ERAS Society also published guidelines for perioperative care after radical cystectomy, in which early removal of a nasogastric tube, controlled fluid administration, and multimodal prevention of ileus, including gum chewing, prevention of PONV, and minimally invasive surgery, were recommended^21^.

Compared to the findings for ORC, RARC is associated with shorter hospital stays and lower medical costs^22–25^. However, no active attempts have been made to shorten hospital stays in Japan, partly because of the Japanese medical expense payment system (diagnosis procedure combination/per-diem payment system)^26^. In this study, the hospital stay was significantly shorter in the RARC/ERAS group. Preoperatively, we could safely omit fasting and mechanical and medical bowel preparation, resulting in shortened preoperative hospital stays. Postoperatively, the decreased incidence of complications including POI also led to shorter hospitalization. The time to the acquisition of stoma care skills determined the length of postoperative hospital stay in most patients.

Along with hospital stay, medical costs were lower in the RARC/ERAS group than in the ORC group. Importantly, the medical cost excluding the operation cost decreased from 964.7 thousand yen for ORC to 655.7 thousand yen for RARC/ERAS, reflecting the shorter hospital stay and lower complication rate during treatment. A meta-analysis comparing ORC, RARC/ICUD, and RARC/extracorporeal urinary diversion in Israel and the United States identified ORC with ERAS and RARC/ICUD with ERAS as the two most cost-effective strategies. Importantly, the ERAS protocol improved cost-effectiveness compared to their parallel non-ERAS counterpart^22^. Further large-scale prospective studies are needed to verify the impact of ERAS on medical costs in Japan.

The limitations of this study included the relatively small number of patients and the retrospective nature. Because this was a comparative study between ORC and RARC/ERAS, the pure effect of ERAS could not be addressed. Prospective randomized studies are necessary to further verify the impact of RARC/ERAS on the management of muscle-invasive bladder cancer in Japan.

In conclusion, RARC/ERAS proved safe for treatment of muscle-invasive bladder cancer. Along with shortened hospital stays and decreased perioperative complications, RARC/ERAS consequently reduced the medical costs.

## Methods

### Patients

The medical records of patients with bladder cancer who underwent ORC (between September 2015 and June 2021) or RARC/ERAS (between July 2021 and March 2023) at NTT Medical Center Tokyo (Tokyo, Japan) were retrospectively reviewed. Patient characteristics, surgical results, hospital stay, and medical costs were analyzed.

### ERAS program

#### Multidisciplinary ERAS team

**Figure 1:**
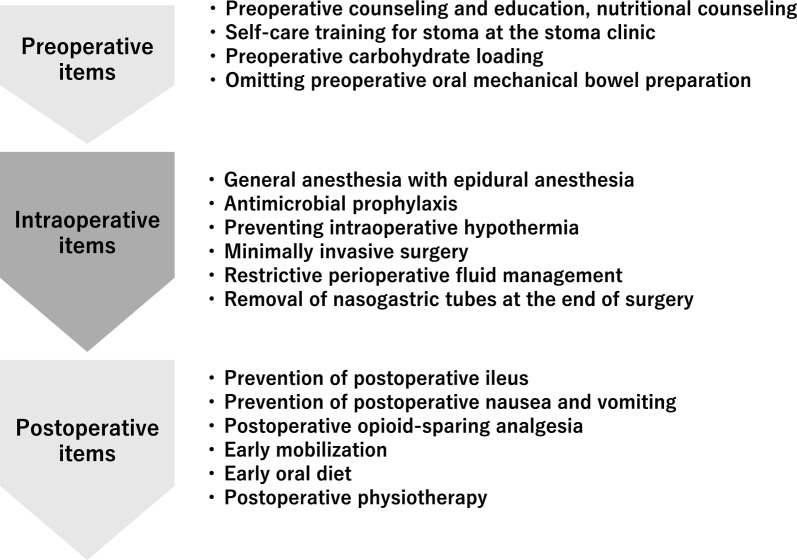
Timeline of ERAS protocol during RARC.

Our ERAS items were provided by a multidisciplinary team composed of urologists, anesthesiologists, physical therapists, registered dietitians, nurses, pharmacists, diabetologists, and clinical care pathway committee members as described elsewhere^27^. The timeline of our ERAS protocol is shown in Fig. 1.

### ERAS items

#### Preoperative items

##### Preoperative counselling and education and nutritional counseling

Preoperative counseling in the outpatient setting was provided by nationally certified nutritionists, urologists, and anesthesiologists. Patients were advised to maintain a normal diet until the night before surgery unless they had malnutrition according to the European Society for Clinical Nutrition and Metabolism guidelines^28^. Patients were given a document on their expected recovery after surgery. Preoperative morbidities were optimized as possible.

Patients were taught self-care for stoma at the stoma clinic before the operation.

##### Preoperative carbohydrate loading

Patients were administered 250 mL of carbohydrate fluid (Arginaid Water®, Nestle healthscience, Tokyo, Japan:100 kcal, 22.5 g carbohydrate with 2.5 g arginine per 125 mL) at 12 and 2 h before surgery.

##### Preoperative oral mechanical bowel preparation

Oral mechanical bowel preparation was omitted. Sennoside (24 mg) was administered before bedtime on the night before surgery.

#### Intraoperative items

##### Anesthesia

General anesthesia was induced using propofol, fentanyl, remifentanil, and rocuronium and maintained using air/oxygen/desflurane, remifentanil, fentanyl, and rocuronium bromide. Epidural anesthesia with 7.5 mg/mL ropivacaine hydrochloride hydrate was provided. The total amount of fentanyl was restricted to 5 μg/kg. Intravenous acetaminophen was administered before the end of surgery.

##### Preventing intraoperative hypothermia

Warming blankets (Full Body Bair Hugger™, 3 M) were used to prevent hypothermia during surgery.

##### Minimally invasive surgery

RARC was performed by experienced urologists certified by the Japanese Urological Association using the Da Vinci Xi® system (Intuitive Surgical Ltd., Sunnyvale, CA, USA). Urinary diversion was performed intracorporeally (intracorporeal urinary diversion [ICUD]). The small intestine was cut and anastomosed using the SureForm® stapling system (Intuitive Surgical Ltd., Sunnyvale, CA, USA).

##### Resection site drainage

A drainage tube (8 mm) was placed at the bottom of the pelvic cavity until postoperative day (POD) 1.

##### Restrictive perioperative fluid management

Intraoperative intravenous fluid administration was limited to 3 mL/kg/h, and it included Ringer’s bicarbonate solution, antibiotics, and acetaminophen.

##### Nasogastric intubation

Nasogastric tubes were removed at the end of surgery.

#### Postoperative items

##### Urethral drainage

A double-lumen urethral catheter (20 Fr) was placed in the neobladder until its removal on POD 14 in patients who underwent ileum neobladder urinary diversion.

##### Prevention of postoperative ileus

To prevent postoperative ileus, patients were encouraged to chew gum every 3 h starting 3 h after surgery. Magnesium oxide (300 mg) was administered three times daily until POD 7.

##### Prevention of postoperative nausea and vomiting

To prevent postoperative nausea and vomiting, 6.6 mg of dexamethasone were intravenously administered at the induction of anesthesia.

##### Postoperative opioid-sparing analgesia

For postoperative analgesia, patients were intravenously administered 1000 mg of acetaminophen every 6 h for 24 h and oral celecoxib (200 mg) every 12 h.

##### Early mobilization

The patients were instructed to stand 3 h after surgery. The physical therapist assessed the patients’ condition and assisted with early mobilization from POD 1. On POD 1, patients were encouraged to walk 100 m and attempt to be out of bed for 6 h. They were also encouraged to be out of bed for more than 2 h. On POD 2, patients walked more than 300 m with assistance by physical therapists. They were encouraged to be out of bed for more than 6 h.

##### Early oral diet

Patients were allowed to drink clear fluid 3 h after surgery. A liquid diet was also provided 3 h after surgery.

### Statistical analysis

Fisher’s chi-squared test was used to analyze categorical variables. Mann-Whitney U test was performed to analyze numerical variables. Statistical significance was indicated by *P* < 0.05. All statistical analyses were performed using SPSS version 24.

### Ethics approval and consent to participate

This study was conducted in accordance with the Declaration of Helsinki (revised in 2013).

This study is approved by the ethics committee of NTT Medical Center Tokyo. Informed consent was obtained in the form of opt-out on the web-site. Those who rejected were excluded.

## Data Availability

The datasets generated during the current study are available from the corresponding author on reasonable request.
